# Differences in acute retroviral syndrome by HIV-1 subtype in a multicentre cohort study in Africa

**DOI:** 10.1097/QAD.0000000000001659

**Published:** 2017-11-09

**Authors:** Eduard J. Sanders, Matthew A. Price, Etienne Karita, Anatoli Kamali, William Kilembe, Linda-Gail Bekker, Shabir Lakhi, Mubiana Inambao, Omu Anzala, Patricia E. Fast, Jill Gilmour, Kimberly A. Powers

**Affiliations:** a Centre for Geographic Medicine Research-Coast, Kenya Medical Research Institute (KEMRI), Kilifi, Kenya; b University of Oxford, Headington, UK; c International AIDS Vaccine Initiative, New York City, New York; d Department of Epidemiology and Biostatistics, University of California, San Francisco, California, USA; e Project San Francisco, Kigali, Rwanda; f Uganda Research Unit on AIDS, Medical Research Council/Uganda Virus Research Institute, Entebbe, Uganda; g Zambia Emory Research Project, Lusaka, Zambia; h Desmond Tutu HIV Centre, University of Cape Town, South Africa; i Kenya AIDS Vaccine Initiative, Nairobi, Kenya; j Department of Epidemiology, Gillings School of Global Public Health, University of North Carolina at Chapel Hill, Chapel Hill, North Carolina, USA.

**Keywords:** acute or early HIV-1 infection, acute retroviral syndrome, HIV-1 *POL* subtype, signs and symptoms

## Abstract

**Objective:**

Symptoms of acute retroviral syndrome (ARS) may be used to identify patients with acute HIV-1 infection who seek care. ARS symptoms in African adults differ by region. We assessed whether reporting of ARS was associated with HIV-1 subtype in a multicentre African cohort study representing countries with predominant HIV-1 subtypes A, C, and D.

**Methods:**

ARS symptoms were assessed in adults enrolling at least 6 weeks after the estimated date of infection in an acute and early HIV-1 infection cohort study. HIV-1 subtype was determined by *POL* genotyping. We used log-binomial regression to compare ARS symptom prevalence among those with subtype A vs. C or D, adjusting for sex, time since enrolment, and enrolment viral load.

**Results:**

Among 183 volunteers ascertained within 6 weeks after estimated date of infection, 77 (42.0%) had subtype A, 83 (45.4%) subtype C, and 23 (12.6%) subtype D infection. Individuals with subtype A were 1.40 (95% confidence interval: 1.17, 1.68) times as likely as individuals with subtypes C or D to report any ARS symptoms; each individual symptom other than rash was also more prevalent in subtype A than in subtype C or D, with prevalence ratios ranging from 1.94 (1.40, 2.70) for headache to 4.92 (2.24, 10.78) for lymphadenopathy.

**Conclusion:**

Individuals with subtype A were significantly more likely than individuals with subtypes C or D to report any ARS symptoms. HIV-1 subtypes may help explain differences in ARS that have been observed across regions in Africa, and may impact the yield of symptom-based screening strategies for acute HIV infection detection.

## Abstract 2

Supplemental Digital Content is available in the text

## Introduction

Prompt identification and treatment of adults newly infected with HIV-1 can dramatically reduce onward transmission and improve the health of the infected individual [1]. Identifying adults with acute HIV-1 infection (AHI), a substantial portion of whom seek urgent care even in resource-constrained settings [2], therefore has tremendous public health importance [3]. Unfortunately, AHI detection has not been emphasized in Sub-Saharan Africa (sSA), where the epidemic burden is greatest [4,5].

Across sSA, AHI symptom prevalence has varied considerably, with higher estimates reported in Kenya [6] than in Uganda [7] and Zambia [8]. Symptoms develop approximately 2 weeks after HIV-1 acquisition, just before plasma viral load peaks [9,10], and the number of symptoms correlates with higher plasma viral load before seroconversion [11]. Thus, strategies aiming to diagnose symptomatic AHI patients at care seeking may identify those with more severe symptoms and higher peak [11] and set point [9,12] viral load.

Although infection with HIV-1 subtypes C or D (vs. subtype A) has predicted faster progression to AIDS and death in several studies in Africa [12–15], little systematic research has been conducted on the clinical manifestations of AHI in relation to subtype. As substantial geographical differences in ARS have been documented in sSA [2,6–8] and HIV-1 subtype varies geographically, we hypothesized that the occurrence of symptoms around the time of seroconversion could be correlated with HIV-1 subtype. We sought to test this hypothesis in the largest seroconverter cohort study from Africa, a research collaboration of nine clinical research centres (CRC), representing countries with predominant HIV-1 subtypes A, C, or D [16].

## Methods

### Study design and procedures

Data derived from ‘protocol C’ of the International AIDS Vaccine Initiative (IAVI), a multicentre early HIV infection cohort study [15]. As described previously [16], adults at risk of HIV-1 were enrolled into a prospective cohort study across CRCs in Kenya, Uganda, Rwanda, Zambia, and South Africa. These HIV incidence cohorts included predominantly: cohabiting heterosexual partners of an HIV infected, antiretroviral therapy-naïve person (Zambia; Rwanda; Uganda); sex workers and their clients, or clients with a sexually transmitted infection (Kenya); MSM (Kenya and Rustenburg, South Africa); or adolescents (Cape Town). Incidence study volunteers were tested for HIV-1 monthly or quarterly (depending on site) as described previously [17,18].

Volunteers with incident HIV infection were invited to enrol into protocol C between February 2006 and December 2011. At enrolment, all patients completed a standardized questionnaire asking whether they had experienced symptoms consistent with acute retroviral syndrome (ARS) in the past 90 days: fever, headache, myalgia/arthralgia, fatigue, anorexia, pharyngitis, diarrhoea, night sweats, skin rash, lymphadenopathy, oral ulcers, or ‘other’. Enrolment viral load [16] was measured and HIV-1 subtype was determined by sequencing the *POL* (HIV-1 genome) region [19].

The ethical review boards of all participating CRCs approved the study protocol and all study volunteers provided written informed consent.

### Estimated date of infection and timing of symptom ascertainment

The estimated date of HIV-1 infection (EDI) was determined as follows [20]: 10 days before the sample collection date when the sample had a positive RNA viral load, negative p24 antigen and negative HIV-1 serology; 14 days before a positive p24 antigen test (regardless of RNA result) with negative HIV-1 serology; 19 days before the date that rapid HIV-1 antibody tests were discordant (regardless of p24 antigen or RNA result); or the mid-term date between a previously negative and subsequently fully positive HIV-1 serologic test (two rapid tests conducted in parallel) [2].

To limit recall error and maximize questionnaire sensitivity, we restricted the current analysis to patients who enrolled in protocol C within 6 weeks of EDI [15], based on our earlier finding that reported symptom prevalence was considerably lower in patients evaluated more than 6 weeks vs. within 6 weeks after EDI [21].

### Data analysis

We first calculated descriptive statistics, including prevalence of each ARS symptom and prevalence of ‘any’ symptom by HIV-1 subtype. We then used two sets of multivariable log-binomial regression models to estimate the association between HIV-1 subtype and reporting any ARS symptom. In one set, we included enrolment log_10_ viral load to estimate the direct effect (i.e. not mediated through viral load) of subtype on ARS symptoms; in the other, we excluded viral load to estimate the total effect of subtype. In both sets, we started with a full model containing two potential confounders – sex and time between EDI and enrolment – that have been reported as being associated with ARS symptoms [21,22] and were associated with subtype in our study (because of differences in population types and enrolment timing across sites). To maximize precision, we dichotomized the exposure in each set of models as subtype A vs. subtype C or D (combined) after we observed that symptom prevalence and other characteristics were similar for subtypes C and D (Supplemental Figure S1, Supplemental Table S1). We used backward selection with α = 0.05 to sequentially remove variables and arrive at a final model within each set.

To explore the possibility that differences in reported symptoms across subtypes might be because of differential symptom reporting/ascertainment and/or unmeasured confounding across study centres, we conducted a sensitivity analysis of ARS symptoms by subtype only among volunteers at the two centres (Masaka and Kilifi), where cases across the three subtypes were enrolled. Multivariable analyses were not possible in this subset. Additionally, we examined univariable associations between 37 common human leucocyte antigen (HLA) variants (see Supplemental List S1) and ARS symptom reporting (any vs. none) to determine (accounting for multiple comparisons) whether adjustment for any of these HLA types was needed. We performed a similar analysis to assess whether calendar year of enrolment was associated with ARS symptom reporting.

## Results

A total of 183 volunteers were ascertained within 6 weeks following EDI, 77 (42%) with subtype A and 106 (58%) with subtype C or D infection (Table 1). Overall, approximately one-third of participants were female, with higher proportions of females among those with subtype C or D. Participants with subtype A were on average 3 years younger than participants with subtype C or D. Most volunteers with subtype A enrolled in Kigali or Kilifi (Table 1), and most with subtype C enrolled in Lusaka or Copperbelt Province (Supplemental Table S1). Participants with subtype D enrolled only in Masaka, Kilifi, and Entebbe (Supplemental Table S1). Of those with known risk group status, persons with subtype A infections were evenly split between MSM and serodiscordant heterosexual couples, whereas most with subtype C or D infections were members of serodiscordant couples. Persons with subtype A infection were enrolled a few days earlier on average and had a slightly higher viral load than did persons with subtypes C or D.

**Table 1:** Volunteer characteristics at enrolment^a^.

Characteristic	Overall (*N* = 183)	Subtype A (*N* = 77)	Subtype C or D (*N* = 106)
*N* (%) female	63 (34.4)	21 (27.3)	42 (39.6)
Median (range) age^b^	29 (16–58)	27 (19–52)	30 (16–58)
Site:^b^			
Kigali	33 (18.0)	29 (37.7)	4 (3.8)
Masaka	22 (12.0)	5 (6.5)	17 (16.0)
Kilifi	47 (25.7)	37 (48.0)	10 (9.4)
Nairobi	5 (2.7)	4 (5.2)	1 (1.0)
Lusaka	46 (25.1)	1 (1.3)	45 (42.5)
Entebbe	4 (2.2)	1 (1.3)	3 (2.8)
Cape Town	3 (1.7)	0 (0.0)	3 (2.8)
Copperbelt	17 (9.3)	0 (0.0)	17 (16.0)
Rustenburg	6 (3.3)	0 (0.0)	6 (5.7)
Number (%) in risk group:^b^			
Serodiscordant couples	120 (65.6)	36 (46.8)	84 (79.3)
MSM	45 (24.6)	35 (45.4)	10 (9.4)
Other/don’t know	18 (9.8)	6 (7.8)	12 (11.3)
Median (interquartile range) days since EDI^b^	25 (19–33)	21 (18–32)	26 (21–33)
Median (range) enrolment log_10_ viral load	5.0 (1.4–7.3)	5.2 (1.4–7.3)	4.9 (2.6–7.0)
Median (range) number of ARS^c^ symptoms per participant ^b^	3 (0, 11)	5 (0, 11)	2 (0,8)

ARS, acute retroviral syndrome; EDI, estimated date of HIV-1 infection.

^a^All within 42 days of estimated infection acquisition date.

^b^Subtype A vs. subtype C or D comparison statistically significant at α = 0.05.

^c^Acute retroviral syndrome.

**Fig. 1:**
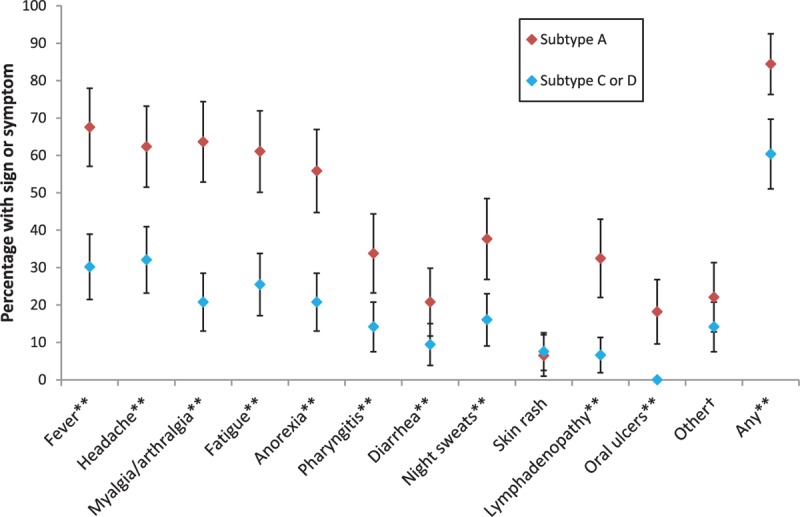
Prevalence of acute retroviral syndrome symptoms by dichotomized HIV-1 subtype, all sites.

^∗∗^Statistically significant at α = 0.05. ^†^Symptoms reported under ‘other’ were gastrointestinal symptoms, ophthalmic symptoms, cough, dizziness, malaise, and rhinitis.

Overall, 84.4% (95% confidence interval: 76.3–92.5%) of subtype A volunteers reported any ARS symptoms, compared with 60.4% (50.1–68.8%) of volunteers with subtypes C or D (Fig. 1). The median (range) number of symptoms per volunteer was 5 (0, 11) for subtype A, 2 (0, 8) for subtype C, and 1 (0, 8) for subtype D. In Kigali and Kilifi, the median was 4 (0, 8) and 7 (0, 11) among their 29 and 37 subtype A volunteers, respectively. The percentage of subtype A participants reporting each of the individual symptoms ranged from 6.5% (1.0–12.0%) for skin rash to 67.5% (57.1–78.0%) for fever, compared with a range of 6.6% (1.9–11.3%) for skin rash to 32.1% (23.2–41.0%) for headache among those with subtypes C or D. Each of the specific symptoms other than rash (unadjusted prevalence ratio = 0.98, 95% confidence interval: 0.32–2.98) was more prevalent in subtype A than in subtype C or D (combined), with the unadjusted prevalence ratio ranging from 1.94 (1.40, 2.70) for headache to 4.92 (2.24, 10.78) for lymphadenopathy. The corresponding prevalence ratio for reporting any symptoms was 1.40 (1.17, 1.68). Findings were similar but less precise in the sensitivity analysis restricted to Kilifi and Masaka volunteers (Supplemental Figure S2).

None of the 37 HLA types we examined was associated with ARS symptoms (results available on request), nor was calendar year of enrolment (*P* = 0.8), so we did not include HLA type or calendar time in our models. In both sets of multivariable models (with and without viral load), neither of the potential confounders (sex, time since enrolment) was selected for the final model. The total effect estimated in the final model was thus identical to the unadjusted prevalence ratio (1.40) above. The prevalence ratio arising from the final model with viral load included was 1.47 (1.20, 1.80). The similarity of this result to the estimate from the model without viral load suggests that the relationship between viral subtype and ARS symptoms is not mediated through viral load.

## Discussion

ARS symptoms were more prevalent in patients infected with HIV-1 subtype A vs. subtype C or D in our multicentre cohort in Africa, a difference that was independent of viral load [6], HLA type, sex, and time of enrolment. These findings, from the largest study of ARS symptoms by HIV-1 subtype to date, may help to explain differences in ARS reporting across previous studies from different regions of sSA. Indeed, the greater number of symptoms per participant that we observed (overall median of 3) vs. that of the recent study of Robb *et al.*[9] (median = 1) may be partially explained by differences in cohort composition according to subtype [23].

Although it is unknown whether subtype-specific viral properties or immune activation cause AHI symptoms, observed symptom differences by HIV-1 subtype have public health significance. Patients with symptomatic AHI frequently seek healthcare [24–26], presenting opportunities for diagnosis and immediate treatment. Unfortunately, guidance is lacking on who should be evaluated for AHI in sSA [5,27], but we previously showed that screening at-risk adults for AHI using a simple algorithm based on seven characteristics would substantially reduce the number of HIV-1-seronegative patients requiring testing [27]. The yield of this algorithm and impact of RNA testing (compared with standard HIV testing) will be assessed among 2875 adults seeking urgent care for symptoms in a proof-of-concept trial in Kenya (R01 AI124968–01A1).

We note that there may have been some subtype misclassification because of our reliance on the *POL* region, and that our insights exclude recombinant subtypes [28] and subtypes other than A, C, or D. Additionally, although most infections in discordant couples were caused by a single strain [29], approximately a third of MSM in Kenya had multiple strains at infection [30], some of which may have been obscured in analyses. Finally, we note that our enrolment viral load was likely a ‘postpeak’ measurement, and the extent to which the relationship between this measure and peak viral load is consistent across subtypes will determine the extent to which our estimated direct effect of subtype reflects the effect not mediated through peak viral load.

Despite these limitations, the differences that we observed in ARS symptoms across HIV-1 subtypes suggest that further investigation of viral characteristics causing immune activation and control is necessary [23]. As studies in East Africa [9] and South Africa [31] demonstrate that median peak viral loads are very high, corresponding with high transmissibility, further research on the yield of symptom-based AHI screening algorithms in different regions of sSA is needed.

## Acknowledgements

We thank staff at the nine clinical research centres participating in this study. Special thanks to Jianming Tang at the University of Alabama for conducting analysis of HLA and ARS symptoms. The KWTRP at the Centre for Geographical Medicine Research, Kilifi is supported by core funding from the Wellcome Trust (#203077/Z/16/Z). This work was partially funded by IAVI with the generous support of USAID and other donors; a full list of IAVI donors is available at www.iavi.org. E.J.S. receives research funding from IAVI, the NIH, and the Wellcome Trust. The contents are the responsibility of the study authors and do not necessarily reflect the views of USAID, the NIH, the United States Government, or the Wellcome Trust. This report was published with permission from KEMRI.

This work was also supported through the Sub-Saharan African Network for TB/HIV Research Excellence (SANTHE), a DELTAS Africa Initiative (grant # DEL-15-006). The DELTAS Africa Initiative is an independent funding scheme of the African Academy of Sciences (AAS)'s Alliance for Accelerating Excellence in Science in Africa (AESA) and supported by the New Partnership for Africa's Development Planning and Coordinating Agency (NEPAD Agency) with funding from the Wellcome Trust (grant # 107752/Z/15/Z) and the UK government.

P.E.F., J.G., and M.A.P. designed the IAVI protocol C, including data instruments for acute retroviral syndrome assessment. E.J.S., E.K., A.K., W.K., L-G.B., S.L., M.I., and O.A. supported data collections in the period 2006–2011. E.J.S. developed the idea for ARS comparisons across HIV-1 subtypes, K.A.P. designed and conducted the statistical analysis, and helped draft and revise the manuscript. All authors reviewed and approved of the report.

### Conflicts of interest

There are no conflicts of interest.

## Supplementary Material

Supplemental Digital Content

## References

[1] DHHS. Panel on Antiretroviral Guidelines for Adults and Adolescents. Department of Health and Human Services. [Accessed 29 September 2015]. Available at http://www.aidsinfo.nih.gov/ContentFiles/AdultandAdolescentGL.pdf: pp. 133–139

[2] Sanders EJ Wahome E Mwangome M Thiong’o AN Okuku HS Price MA Most adults seek urgent healthcare when acquiring HIV-1 and are frequently treated for malaria in coastal Kenya. *AIDS* 2011; 25:1219–1224. 21505300 10.1097/QAD.0b013e3283474ed5

[3] Powers KA Cohen MS Acute HIV-1 infection in sub-Saharan Africa: a common occurrence overlooked. *AIDS* 2014; 28:1365–1367. 24959964 10.1097/QAD.0000000000000277

[4] Sharma M Ying R Tarr G Barnabas R Systematic review and meta-analysis of community and facility-based HIV testing to address linkage to care gaps in sub-Saharan Africa. *Nature* 2015; 528:S77–S85. 26633769 10.1038/nature16044 PMC4778960

[5] Prins HA Mugo P Wahome E Mwashigadi G Thiong’o A Smith A Diagnosing acute and prevalent HIV-1 infection in young African adults seeking care for fever: a systematic review and audit of current practice. *Int Health* 2014; 6:82–92. 24842982 10.1093/inthealth/ihu024 PMC4049276

[6] Lavreys L Thompson ML Martin HL Jr Mandaliya K Ndinya-Achola JO Bwayo JJ Primary human immunodeficiency virus type 1 infection: clinical manifestations among women in Mombasa, Kenya. *Clin Infect Dis* 2000; 30:486–490. 10722432 10.1086/313718

[7] Morgan D Mahe C Whitworth J Absence of a recognizable seroconversion illness in Africans infected with HIV-1. *AIDS* 2001; 15:1575–1576. 11504991 10.1097/00002030-200108170-00016

[8] Sullivan PS Fideli U Wall KM Chomba E Vwalika C Kilembe W Prevalence of seroconversion symptoms and relationship to set-point viral load: findings from a subtype C epidemic, 1995–2009. *AIDS* 2012; 26:175–184. 22089380 10.1097/QAD.0b013e32834ed8c8 PMC3589587

[9] Robb ML Eller LA Kibuuka H Rono K Maganga L Nitayaphan S Prospective study of acute HIV-1 infection in adults in East Africa and Thailand. *N Engl J Med* 2016; 374:2120–2130. 27192360 10.1056/NEJMoa1508952 PMC5111628

[10] Lindback S Karlsson AC Mittler J Blaxhult A Carlsson M Briheim G Viral dynamics in primary HIV-1 infection. Karolinska Institutet Primary HIV Infection Study Group. *AIDS* 2000; 14:2283–2291. 11089616 10.1097/00002030-200010200-00009

[11] Lavreys L Baeten JM Overbaugh J Panteleeff DD Chohan BH Richardson BA Virus load during primary human immunodeficiency virus (HIV) type 1 infection is related to the severity of acute HIV illness in Kenyan women. *Clin Infect Dis* 2002; 35:77–81. 12060878 10.1086/340862

[12] Lavreys L Baeten JM Chohan V McClelland RS Hassan WM Richardson BA Higher set point plasma viral load and more-severe acute HIV type 1 (HIV-1) illness predict mortality among high-risk HIV-1-infected African women. *Clin Infect Dis* 2006; 42:1333–1339. 16586394 10.1086/503258

[13] Kiwanuka N Laeyendecker O Robb M Kigozi G Arroyo M McCutchan F Effect of human immunodeficiency virus type 1 (HIV-1) subtype on disease progression in persons from Rakai, Uganda, with incident HIV-1 infection. *J Infect Dis* 2008; 197:707–713. 18266607 10.1086/527416

[14] Baeten JM Chohan B Lavreys L Chohan V McClelland RS Certain L HIV-1 subtype D infection is associated with faster disease progression than subtype A in spite of similar plasma HIV-1 loads. *J Infect Dis* 2007; 195:1177–1180. 17357054 10.1086/512682

[15] Amornkul PN Karita E Kamali A Rida WN Sanders EJ Lakhi S IAVI Africa HIV Prevention Partnership. Disease progression by infecting HIV-1 subtype in a seroconverter cohort in sub-Saharan Africa. *AIDS* 2013; 27:2775–2786. 24113395 10.1097/QAD.0000000000000012 PMC3815107

[16] Kamali A Price MA Lakhi S Karita E Inambao M Sanders EJ Creating an African HIV Clinical Research and Prevention Trials Network: HIV prevalence, incidence and transmission. *PLoS One* 2015; 10:e0116100. 25602351 10.1371/journal.pone.0116100 PMC4300215

[17] Sanders EJ Okuku HS Smith AD Mwangome M Wahome E Fegan G High HIV-1 incidence, correlates of HIV-1 acquisition, and high viral loads following seroconversion among MSM. *AIDS* 2013; 27:437–446. 23079811 10.1097/QAD.0b013e32835b0f81 PMC3929859

[18] Price MA Rida W Mwangome M Mutua G Middelkoop K Roux S Identifying at-risk populations in Kenya and South Africa: HIV incidence in cohorts of men who report sex with men, sex workers, and youth. *J Acquir Immune Defic Syndr* 2012; 59:185–193. 22227488 10.1097/QAI.0b013e31823d8693

[19] Price MA Wallis CL Lakhi S Karita E Kamali A Anzala O Transmitted HIV type 1 drug resistance among individuals with recent HIV infection in East and Southern Africa. *AIDS Res Hum Retroviruses* 2011; 27:5–12. 21091377 10.1089/aid.2010.0030 PMC3045073

[20] Fiebig EW Wright DJ Rawal BD Garrett PE Schumacher RT Peddada L Dynamics of HIV viremia and antibody seroconversion in plasma donors: implications for diagnosis and staging of primary HIV infection. *AIDS* 2003; 17:1871–1879. 12960819 10.1097/00002030-200309050-00005

[21] Sanders EJ Powers KA Karita E Kamali A Kilemba W Allen S Recall of acute retroviral symptoms in a multicentre cohort study in Africa. *AIDS Res Hum Retroviruses* 2014; 30 Suppl 1:A93.

[22] McKellar MS Cope AB Gay CL McGee KS Kuruc JD Kerkau MG Acute HIV-1 infection in the Southeastern United States: a cohort study. *AIDS Res Hum Retroviruses* 2013; 29:121–128. 22839749 10.1089/aid.2012.0064 PMC3537297

[23] Hassan A Hare J Price MA Bjorkman P Allen S Gilmour J Few Acute HIV-1 Symptoms and High Set-point Viral Load in Subtype C infections. Conference on Retroviruses and Opportunistic Infections (CROI) 2017,#202:Seattle, Washington

[24] Bebell LM Pilcher CD Dorsey G Havlir D Kamya MR Busch MP Acute HIV-1 infection is highly prevalent in Ugandan adults with suspected malaria. *AIDS* 2010; 24:1945–1952. 20543656 10.1097/QAD.0b013e32833bb732 PMC2909782

[25] Sanders EJ Mugo P Prins HA Wahome E Thiong’o AN Mwashigadi G Acute HIV-1 infection is as common as malaria in young febrile adults seeking care in coastal Kenya. *AIDS* 2014; 28:1357–1363. 24556872 10.1097/QAD.0000000000000245 PMC4032215

[26] Pastor L Parker E Carrillo J Urrea V Fuente-Soro L Respeito D A cytokine pattern that differentiates preseroconversion from postseroconversion phases of primary HIV infection. *J Acquir Immune Defic Syndr* 2017; 74:459–466. 28225519 10.1097/QAI.0000000000001272

[27] Sanders EJ Wahome E Powers KA Werner L Fegan G Lavreys L Targeted screening of at-risk adults for acute HIV-1 infection in sub-Saharan Africa. *AIDS* 2015; 29 Suppl 3:S221–S230. 26562811 10.1097/QAD.0000000000000924 PMC4714928

[28] Palm AA Esbjornsson J Mansson F Kvist A Isberg PE Biague A Faster progression to AIDS and AIDS-related death among seroincident individuals infected with recombinant HIV-1 A3/CRF02_AG compared with sub-subtype A3. *J Infect Dis* 2014; 209:721–728. 23935204 10.1093/infdis/jit416

[29] Carlson JM Schaefer M Monaco DC Batorsky R Claiborne DT Prince J HIV transmission. Selection bias at the heterosexual HIV-1 transmission bottleneck. *Science* 2014; 345:1254031. 25013080 10.1126/science.1254031 PMC4289910

[30] Macharia G Yue L Dilernia D El-Badry E McGowan E Price M Transmission of multiple HIV-1 founder viruses with high level of recombination in MSM in Kenya. R4P Conference 2016, abstract 499: Chicago, USA

[31] Ndhlovu ZM Kamya P Mewalal N Kloverpris HN Nkosi T Pretorius K Magnitude and kinetics of CD8+ T cell activation during hyperacute HIV infection impact viral set point. *Immunity* 2015; 43:591–604. 26362266 10.1016/j.immuni.2015.08.012 PMC4575777

