# Supplementary material for: Differences in acute retroviral syndrome by HIV-1 subtype in a multicentre cohort study in Africa
Source: AIDS. 2017 Nov 9;31(18):2541–6. doi: 10.1097/QAD.0000000000001659 (PMC5690309; doi:10.1097/QAD.0000000000001659)
Supplement: Supplemental Digital Content [file aids-31-2541-s001.doc]

**Supplemental List S1. HLA Variants Examined**

A01, A02, A03, A23, A29, A30, A33, A34, A36, A66, A68, A74, B07, B14, B1503, B1510, B15x*, B18, B35, B42, B44, B45, B49, B53, B57, B5801, B5802, B81, C02, C03, C04, C06, C07, C08, C16, C17, C18, A6802, B1302, DRB13023, DQB03023

* B15x = B1501, B1516, B1517, B1531, B1547, B1583

**Supplemental Figure S1. Prevalence of ARS symptoms by HIV-1 subtype, all sites**

** A vs. C and A vs. D statistically significant at α=0.05 * A vs. C, but not A vs. D, statistically significant at α=0.05

† Symptoms reported under “other” were GI symptoms, ophthalmic symptoms, cough, dizziness, malaise, and rhinitis.

**Supplemental Figure S2. Prevalence of ARS symptoms by dichotomized HIV-1 subtype, Masaka & Kilifi**

** Statistically significant at α=0.05

† Symptoms reported under “other” were GI symptoms, ophthalmic symptoms, cough, dizziness, malaise, and rhinitis.

Supplemental Table S1. Volunteer Characteristics at Enrollment*

| **Characteristic** | **Overall (N=183)** | **Subtype A (N=77)** | **Subtype C (N=83)** | **Subtype D (N=23)** |
| --- | --- | --- | --- | --- |
| N (%) female | 63 (34.4) | 21 (27.3) | 33 (39.8) | 9 (39.1) |
| Median (range) age ^†^ | 29 (16-58) | 27 (19-52) | 30 (16-52) | 29 (17-58) |
| Site: ^† ‡ **^ |  |  |  |  |
| Kigali | 33 (18.0) | 29 (37.7) | 4 (4.8) | 0 (0.0) |
| Masaka | 22 (12.0) | 5 (6.5) | 2 (2.4) | 15 (65.2) |
| Kilifi | 47 (25.7) | 37 (48.0) | 5 (6.0) | 5 (21.8) |
| Nairobi | 5 (2.7) | 4 (5.2) | 1 (1.2) | 0 (0.0) |
| Lusaka | 46 (25.1) | 1 (1.3) | 45 (54.2) | 0 (0.0) |
| Entebbe | 4 (2.2) | 1 (1.3) | 0 (0.0) | 3 (13.0) |
| Cape Town | 3 (1.7) | 0 (0.0) | 3 (3.6) | 0 (0.0) |
| Copperbelt | 17 (9.3) | 0 (0.0) | 17 (20.5) | 0 (0.0) |
| Rustenburg | 6 (3.3) | 0 (0.0) | 6 (7.2) | 0 (0.0) |
| Number (%) in risk group: ^† ‡ **^ |  |  |  |  |
| Serodiscordant couples | 120 (65.6) | 36 (46.8) | 68 (81.9) | 16 (69.6) |
| Men reporting sex with men | 45 (24.6) | 35 (45.4) | 5 (6.0) | 5 (21.7) |
| Other / don’t know | 18 (9.8) | 6 (7.8) | 10 (12.1) | 2 (8.7) |
| Median (IQR) days since EDI ^†^ | 25 (19-33) | 21 (18-32) | 26 (21-32) | 25 (19-34) |
| Median (range) log_10_ viral load | 5.0 (1.4-7.3) | 5.2 (1.4-7.3) | 4.9 (2.6-7.0) | 5.0 (3.5-6.7) |
| Median (range) number of ARS^¶^ symptoms per participant^† ‡^ | 3 (0, 11) | 5 (0, 11) | 2 (0, 8) | 1 (0, 8) |

***** all within 42 days of estimated infection acquisition date

^†^ Subtype A vs subtype C comparison statistically significant at α = 0.05

^‡^ Subtype A vs subtype D comparison statistically significant at α = 0.05

^**^ Subtype C vs subtype D comparison statistically significant at α = 0.05

¶ Acute retroviral syndrome
